# Who is worthy of protection? Revisiting a theoretical model on the social origins of health inequities during the COVID-19 pandemic

**DOI:** 10.1177/14034948261415806

**Published:** 2026-02-08

**Authors:** Karl Gauffin

**Affiliations:** 1 Department of Public Health Sciences, Stockholm University, Sweden; 2 Centre for Health Equity Studies (CHESS), Stockholm University, Sweden

**Keywords:** **s**ocial inequality, COVID-19, Finn Diderichsen, Nancy Fraser, social justice, recognition, redistribution

## Abstract

**Aims:**

This article examines how the Diderichsen model has been used and adapted in research on health inequalities during COVID-19, and explores how the pandemic has prompted further theoretical development. This review therefore addresses the question of how a well-established theoretical framework has helped researchers understand pandemic-related health inequalities and what opportunities exist for its continued refinement.

**Methods:**

A narrative literature review was conducted using Google Scholar, Web of Science, PubMed and Scopus. Included studies cited a key publication presenting the Diderichsen model and addressed COVID-19 as a central topic. After screening 298 articles, 24 were included for full analysis. The studies were categorised by how they engaged with the model – conceptually, empirically or through further development.

**Results:**

The Diderichsen model was commonly used to frame discussions of health inequality or to interpret pandemic-related disparities in exposure, vulnerability and outcomes. Several studies emphasised occupational and housing-related exposure, class-based comorbidities and the unequal social consequences of COVID-19. A smaller number of studies proposed expanded frameworks, incorporating multilevel and temporal dimensions and introducing new mechanisms related to pandemic responses. These adaptations often focused on migrants, ethnic minorities and other particularly affected groups.

**Conclusions:**

**The review confirms the ongoing relevance of the Diderichsen model in pandemic health inequality research. It argues that the model can be further strengthened by explicitly incorporating concepts of political decision-making, symbolic recognition and social justice. This would improve its capacity to capture the full complexity of health inequalities in times of crisis.**

## Introduction

**Figure 1.**
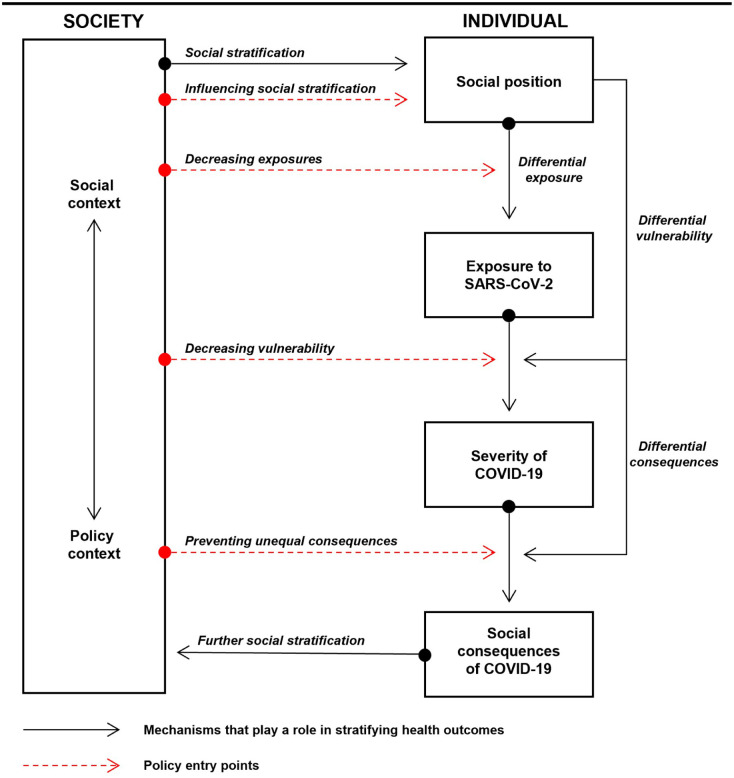
An adapted version of the Diderichsen model.

What have been the most significant contributions of the Nordic countries to research on health inequality? Many would likely highlight the unique, large-scale social epidemiological studies enabled by the Nordic population registers [1]. Others might focus on the ongoing debate about the Scandinavian welfare model and the paradox that health inequality is not necessarily less pronounced in these relatively egalitarian countries compared with, for example, Southern European nations [2]. This text will highlight another contribution of a theoretical nature – namely, the model presented in various versions by Finn Diderichsen and colleagues [3]. Similar arguments have been made in previous review articles that emphasise the fact that many conceptual models of the social determinants of health originate from the Nordic countries [4]. Among these, the model presented by Diderichsen and colleagues is arguably one of the most widely adopted. The model derives from the assumption that health inequality arises through the interplay between unequal exposure and unequal vulnerability, leading to various social and economic consequences that in turn reinforce social inequality (Figure 1).

According to the model, differences in health based on social class, gender or country of origin are often related to differences in *exposure* to a health hazard – such as smoking, unfavourable working conditions or an infectious pathogen. In addition, differences in *vulnerability* might explain why different groups develop different health outcomes despite similar exposures (e.g. to alcohol or physical exertion). Vulnerability may be understood as a combination of susceptibility and capacity to respond to an adverse exposure. Epidemiologically, the relationship between the exposure and health outcome can be disentangled in at least four different ways, depending on mediation and interaction. The exposure might have a direct effect on the outcome, independent of other factors, but it might also shape people’s level of exposure to other harmful conditions, influence how strongly those exposures affect them, or operate through a combination of these pathways [5]. Finally, the resulting health problems can have unequal *social and economic consequences*, depending on factors such as type of employment, insurance, social networks and welfare state arrangements. The model also identifies these interacting chains of disadvantage as potential ‘policy entry points’. Some policies aim to address the root causes by reducing social inequalities themselves, for example, through labour market regulation, taxation, education, and welfare reforms. Others target more immediate mechanisms, such as reducing unequal adverse exposures or diminishing differences in vulnerability to those exposures. Finally, policy can act to mitigate the unequal social and economic consequences of ill health, thereby helping to prevent a further deepening of inequality. Since its introduction, the model has served as an interpretative framework for a growing number of empirical studies aiming to understand the mechanisms behind health inequalities. Alongside other central frameworks on the social determinants of health, the model by Diderichsen and colleagues has had a lasting influence on health inequality research over the past 25 years. It has been cited by more than 1000 studies and features prominently in World Health Organization (WHO) documents on conceptual models advancing our understanding of the social determinants of health [6].

One weakness of the model is that it has never really had a compelling name. The title ‘A Framework for Understanding Social Origins of Health Inequities’ is general and somewhat unwieldy, which is why research usually refers to it simply as ‘the Diderichsen model’. Another potential critique is that the model does not explicitly address the role of historical context, political economy or the ways in which time and place shape population health. When presenting the framework, the authors make repeated references to the concept of social justice or fairness [3]. They refer to those inequalities that result from ‘modifiable social arrangements’ as ‘unfair’. Yet, the central analytical categories of the model – exposure and vulnerability – capture factors that do not necessarily stem from these social arrangements. While the authors do acknowledge the normative dimension of health inequalities by referring to questions of fairness, the model itself does not explicitly define what constitutes social injustice, nor how such injustice contributes to the production and intensification of health inequalities. This raises broader conceptual considerations: how should social injustice be understood in the context of health inequality? In what ways do unjust social structures shape patterns of exposure and vulnerability? And how might a theoretical model more explicitly incorporate injustice as an analytical category, rather than as an implicit background concern? These questions serve as a point of departure for connecting the Diderichsen model to wider debates on social justice and health.

The interplay between individual biology, social environment, exposure and vulnerability has also been addressed in other models. Epidemiologist Nancy Krieger suggests, through her ecosocial theory, that social inequalities are embodied over time as structured social conditions shape exposure, susceptibility and the biological expression of risk, echoing the pathways highlighted in the model suggested by Diderichsen and colleagues [7]. The theory of fundamental causes, by Jo Phelan and Bruce Link, argues that socioeconomic conditions are persistent root causes of health inequalities because they affect access to flexible resources that can protect health across changing risks and contexts [8]. Turning to infectious diseases, the so-called ‘epidemiological triad’ highlights the dynamic interaction between agent, host and environment, reminding us that biological susceptibility, social conditions and contextual factors together determine patterns of infection and disease outcomes [9]. Furthermore, if pandemic health inequalities are seen as a form of social injustice, it may be useful to move beyond traditional models of social determinants of health and consider theories that examine how inequalities are sustained through factors that are clearly modifiable by social arrangements, as Diderichsen and colleagues put it. Philosopher and political theorist Nancy Fraser offers a widely cited framework for understanding social justice, identifying two key dimensions of injustice: economic and cultural, and discusses how these can be addressed through two political remedies: economic redistribution and cultural recognition [10]. Economic redistribution aims to reduce material inequalities, for example, through taxation, welfare policy or financial support, and is most clearly exemplified by social class. Cultural recognition addresses injustices rooted in sociocultural devaluation rather than economic disadvantage. These might include discrimination, stigma, restricted rights and violence. Fraser illustrates this with the example of sexual minorities, who exist across all social classes but are united by their experience of deviating from the heterosexual norm. While analytically distinct, Fraser stresses that these dimensions are deeply interconnected. Categories such as gender and race are shaped by both economic disadvantage and cultural devaluation, as seen in the experiences of women, ethnic minorities, Indigenous populations and people of colour.

## Understanding the unequal pandemic

When the COVID-19 pandemic struck, researchers specialising in health inequality quickly observed that the disease would not affect the world’s population in the same way. Despite early notions of the pandemic as ‘the great equalizer’ [11] and some initial empirical studies [12] showing that higher socioeconomic position was associated with earlier COVID-19 exposure, it was later found that over time COVID-19 related infections and deaths contributed to and exacerbated existing social inequalities in health [13]. The explanations for these findings varied. In Swedish news reports, for instance, high infection rates among the middle class during the early phase of the pandemic were partly attributed to transmission among Alpine travellers in February 2020 [14]. Consequently, income-related disparities in severe COVID-19 cases were relatively low during the first wave but widened considerably during the second wave, which hit Europe in the autumn and winter of 2020–2021 [15]. Since then, numerous studies have confirmed that the pandemic did not affect all groups equally in terms of infection rates, severe illness and death. COVID-19 posed a disproportionately severe threat to the health of the working class [16], including those in precarious employment [17], marginalised immigrants [18], racial minorities [19], incarcerated populations [20] and the homeless [21]. In light of this, the pandemic has increasingly been examined through a health equity lens, with early editorials and opinion pieces applying the Diderichsen model to highlight the mechanisms through which these inequalities were produced and exacerbated, while also referring to the policy entry points as opportunities to prevent the deepening of existing disparities and mitigate future health inequalities [22]. The inclusion of policy entry points makes clear that the framework also carries a normative dimension: it does not merely describe how inequalities arise, partly as a result of social injustices, but also implies that they can and should be addressed through targeted interventions.

Building on this perspective, the present review aims to map and analyse how the Diderichsen model has been applied in COVID-related empirical research and to discuss how the pandemic can serve as a starting point for further development of the theoretical model. The review is guided by three research questions:

1. How is the Diderichsen model used, discussed and applied in theoretical and empirical research about COVID-19?
2. How has the model been further developed based on the experience of the pandemic?
3. How can further developed models give consideration to social justice during a pandemic?

## Methods

Narrative literature reviews serve as a critical synthesis of existing research, offering an overarching perspective on a particular topic. Unlike systematic reviews, which follow a rigid, predefined methodology to minimise bias and ensure reproducibility, narrative reviews are more flexible, allowing for interpretative analysis and contextual understanding. They are particularly useful for summarising broad topics, exploring theoretical developments and identifying gaps in the literature [23]. While systematic reviews prioritise objective evidence synthesis through structured data collection and analysis (e.g. PRISMA guidelines), narrative reviews emphasise subjective interpretation and critique [24]. A narrative review is well-suited for investigating the use of the Diderichsen model in COVID-19 research owing to the need for a broad and interpretative synthesis of how this theoretical framework has been applied across different studies. The narrative synthesis enables an examination of not only the extent to which the Diderichsen model has been employed, but also the nuances of its adaptation, strengths and limitations in explaining health inequities during the pandemic.

### Search strategy and selection criteria

This literature review included peer-reviewed, English-language articles published in scientific journals that met two additional criteria: they cited one of the key publications presenting the Diderichsen model and they treated COVID-19 as a central theme of the study.

Four such key publications by Diderichsen and colleagues were identified and earmarked in the search engines Google Scholar, Web of Science, PubMed and Scopus [3,5,25,26]. Using the search terms ‘covid’, ‘SARS-CoV-2’ or ‘coronavirus’ among articles citing any of these four studies, a set of relevant studies was selected for further review. While there were no restrictions on publication date, all identified articles were published in 2020 or later. The search was conducted on 25 March 2025. An Excel spreadsheet was created and iteratively refined to capture information relevant to the study’s research questions, as well as characteristics necessary for quality assessment and descriptive analysis. The dimension of social inequality addressed in the studies was identified (e.g. class, neighbourhood-level deprivation, ethnicity, income, etc.) as were the types of health outcome. Each study was classified based on how it engaged with the Diderichsen model: (i) model mentioned in the introduction; (ii) model mentioned in the discussion; (iii) model used to interpret empirical findings; (iv) model further developed. Studies could be assigned to one or more of these categories.

## Results

**Figure 2.**
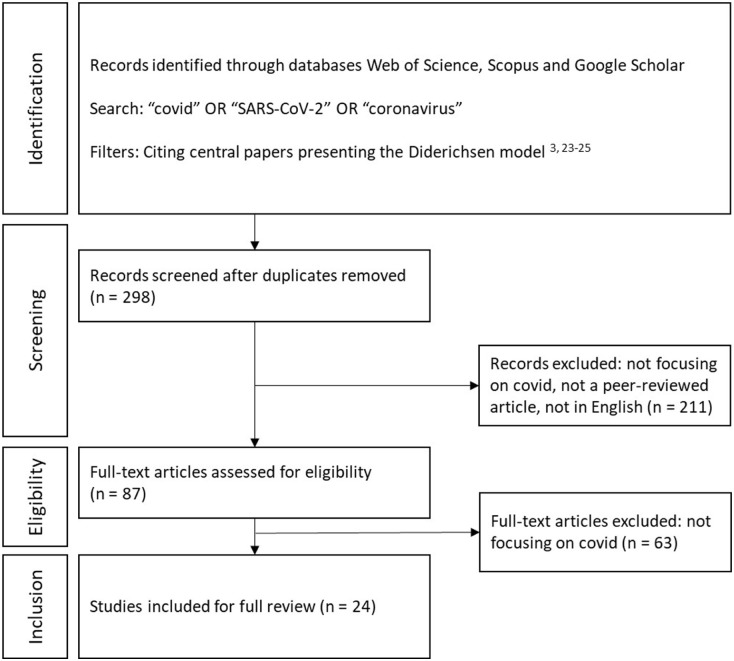
Flow diagram of literature search.

Of a total of 1457 studies that cited one of the four articles presenting the Diderichsen model, 298 studies contained the search terms. The abstracts of all these articles were reviewed, after which duplicates and articles that did not meet the eligibility criteria were excluded. The remaining 87 articles underwent a full-text screening, leading to the exclusion of an additional 63 articles. The primary reason for exclusion at this stage was that the COVID-19 pandemic was only mentioned marginally and the article was actually focused on another public health issue (Figure 2).

### Description of studies

The majority of the 24 included studies were quantitative studies (43%), followed by qualitative studies and reviews (each 22%) and essays (17%). The studies addressed a wide range of inequalities, including income, neighbourhood deprivation, educational level, employment conditions, ethnicity and minority status. A vast majority of the studies focused on COVID-19 related outcomes, such as infection rates or COVID related mortality. Almost half of the studies were conducted in a European country, about 35% referred to multiple countries or a global context and the rest were conducted in other countries, including Brazil, India and the United States (Table I) [27–49].

**Table I.** Included studies.

No.	Authors	Year	Study design	Study setting	Dimension of inequality		Application of Diderichsen model			
					Social dimension	Health dimension	Presented in introduction	Used in discussion	Empirical use of model	Development of model
1	Antequera et al. [27]	2021	Review	Global	Multiple	COVID mortality and morbidity	Yes	No	No	No
2	den Broeder et al. [28]	2022	Essay	Netherlands and UK	Class, neighbourhood	General health	Yes	No	No	No
3	Coleman et al. [59]	2023	Review	UK	Ethnicity	COVID infection and mortality	Yes	Yes	Yes	No
4	Demenech et al. [29]	2020	Quantitative	Brazil	Income	COVID infection and mortality	No	Yes	No	No
5	Diderichsen et al. [30]	2021	Quantitative	Brazil	Education	Disability, diabetes, depression	Yes	Yes	Yes	No
6	Diderichsen [31]	2021	Essay	Sweden	Multiple	COVID mortality	No	Yes	No	No
7	Foster et al. [32]	2020	Quantitative	UK	Socioeconomic	COVID mortality and hospitalisation	Yes	No	No	No
8	Green et al. [33]	2023	Quantitative	UK	Family structure	Psychiatric distress	Yes	No	No	No
9	Hanson et al. [34]	2023	Qualitative	UK	N/A	Salutogenic factors	No	Yes	No	No
10	Hintermeier et al. [35]	2024	Review	Global	Migrant status	COVID infection, hospitalisation, ICU, death	Yes	Yes	Yes	Yes
11	Irizar et al. [36]	2024	Review	Global	Ethnicity	COVID infection, hospitalisation, mortality	Yes	Yes	Yes	Yes
12	Katikireddi et al. [49]	2021	Essay	Global	Ethnicity	COVID mortality and morbidity	Yes	Yes	Yes	Yes
13	Lingam and Suresh Sapkal [37]	2020	Quantitative	India	Income	COVID infection and mortality	Yes	No	No	No
14	Marra et al. [38]	2022	Quantitative	Italy	Socioeconomic	COVID infection, hospitalisation, ICU, mortality and all-cause mortality	Yes	No	No	No
15	McGowan and Bambra [39]	2024	Review	Global	Area-level deprivation	COVID mortality	Yes	Yes	No	No
16	Netrdová et al. [40]	2025	Quantitative	Czechia	Region	All-cause mortality	Yes	Yes	No	Yes
17	Pattanshetty et al. [41]	2022	Quantitative	Global	Degree of conflict affectedness	Vaccination status	No	Yes	No	No
18	Paykani and Oana [42]	2023	Qualitative	OECD	Trust in state capacity influenced by other structural factors	COVID mortality	Yes	No	No	No
19	Rigó and Weyers [43]	2024	Quantitative	Germany	Multiple	Child motor development	Yes	No	No	No
20	Sandholdt et al. [44]	2022	Qualitative	Denmark	Neighbourhood disadvantage	Health promotion intervention	Yes	No	No	No
21	Larsen et al. [45]	2022	Qualitative	Denmark	N/A	Emotional risk	No	Yes	No	No
22	Schrecker [46]	2023	Essay	Global	Multiple	General health	Yes	No	No	No
23	Vignola et al. [47]	2024	Qualitative	USA	Precarious employment	Health risks	Yes	Yes	No	No
24	Ying et al. [48]	2022	Quantitative	USA	Socioeconomic	COVID infection	Yes	No	No	No

OECD: Organisation for Economic Co-operation and Development; ICU: intensive care unit

### The Diderichsen model in research on COVID-19

All studies referred to the Diderichsen model in the introduction or discussion, with a minority explicitly using the model to interpret empirical results or developing a revised version. There were some recurring patterns in the use of the model in the included studies, with three main applications: describing health inequalities; analysing pandemic exposure, vulnerability and consequences; and developing the conceptual model.

#### Model used to describe health inequalities during a pandemic

Several studies referred to the articles presenting the Diderichsen model to illustrate health inequality in broad terms [27,28,45,48]. In these studies, the model is not central but appears rather as a peripheral reference. In such cases, Diderichsen and colleagues are primarily cited in the introduction. The model is used to underscore the relevance of structural and systemic factors in shaping health outcomes, highlighting the broader scholarly consensus that health is not solely determined by individual behaviours or biomedical factors, but is deeply rooted in social conditions. Citing the Diderichsen model serves a strategic function by anchoring the study within the established literature on health inequalities. This helps to position the research as part of a larger academic conversation and to demonstrate theoretical awareness of the field’s foundational concepts.

Several studies interpreted COVID-19 not merely as a pandemic, but as a syndemic – a crisis in which the virus interacted with and intensified existing non-communicable diseases and harmful social conditions, particularly among disadvantaged populations [30,34,39]. In this view, COVID-19 did not create new vulnerabilities but exposed and exacerbated long-standing inequalities shaped by poverty and social exclusion. Similar to the Diderichsen model, the syndemic perspective draws attention to how structural deprivation produces multiple, compounding risk factors for severe illness and mortality. It also underscores the unintended social consequences of pandemic control measures such as lockdowns, which often hit hardest in communities already burdened by poor health and social disadvantage. These insights reinforce the need to understand COVID-19 not only as a biomedical event but as a socially patterned crisis with deeply unequal effects.

#### Model used to analyse pandemic exposures, vulnerabilities and social consequences

The most common way to use the model is to refer to its division into exposure, vulnerability and social consequences. These mechanisms are then presented as part of a framework used to explain why certain groups were more severely affected by the pandemic than others. In this context, *exposure* typically refers to differences in the duration, intensity, and frequency of contact with the SARS-CoV-2 virus. Differences in exposure were frequently associated with occupational roles, particularly in terms of the ability to maintain social distancing at work [29,37]. For example, the fact that infection rates were higher among preschool teachers, supermarket clerks, taxi drivers and dental assistants than university lecturers, lawyers or white-collar public servants can be explained by their more limited opportunities to work remotely or practise social distancing, which in turn increased their exposure to the SARS-CoV-2 virus. Other studies on exposure highlight differences in living environments, where overcrowding limited the ability to isolate sick household members [35].

*Vulnerability*, in contrast, was shaped by a combination of biological and social disadvantages that increased the risk of severe illness from COVID-19. While age was a significant factor, vulnerability was also influenced by class-related comorbidities and, later in the pandemic, by social disparities in vaccination status. Differential vulnerability was often linked to pre-existing chronic conditions, which were more common among disadvantaged groups [38]. Studies described these conditions in terms of their weakening effect on immune function and the heightened risk of severe outcomes from COVID-19 [37]. Additionally, social determinants such as food insecurity, poor nutritional quality, elevated psychological stress and limited access to healthcare services further reduced individuals’ capacity to protect themselves and adhere to public health measures such as lockdowns and social distancing [29]. Also, going beyond the individual level, the study by Netrdová and colleagues applies the concept of vulnerability to the analysis of geographical differences, demonstrating how cultural, political and institutional factors may impact and amplify regional vulnerabilities [40].

Finally, several studies highlight the unequal *social consequences* of COVID-19, emphasising how different groups were more or less equipped to cope with the pandemic’s broader societal and economic impacts, including unemployment, financial hardship and social exclusion [33,37,38]. Differential consequences extended beyond immediate illness to encompass indirect effects such as increased mortality due to disrupted access to medications and medical treatment for pre-existing conditions; both communicable diseases (e.g. tuberculosis, HIV) and non-communicable diseases (e.g. cancer, kidney disease, hypertension, diabetes). Other unmet health needs included mental health services, contraception, institutional deliveries, immunisation, nutritional support and access to abortion services. Additionally, some individuals, particularly women and children, faced heightened risks of domestic violence and abuse during lockdowns. These compounded challenges led to rising mortality, food insecurity, deepening poverty and an overall decline in well-being. Groups with less social capital and fewer resources for prevention and treatment were especially vulnerable to these cascading effects. It is important to note that the findings presented in this section reflect only a subset of studies that explicitly engaged with the Diderichsen model. As such, they do not capture the full breadth of pandemic inequality research but, rather, illustrate how this particular framework has been applied to analyse mechanisms of exposure, vulnerability and social consequences during COVID-19.

#### Development of an adapted model on health inequity during a pandemic

Three articles present an expanded and adapted version of the Diderichsen model, with a specific focus on health inequality during the COVID-19 pandemic. In their systematic literature review, Hintermeier and colleagues [35] introduce a tailored framework based on the Diderichsen model and empirical studies on COVID-19 among migrants, refugees and internally displaced persons. The model differentiates between ‘exposure’, ‘risk’ and ‘impact of COVID-19 measures’. These factors are categorised across three levels: the micro/individual level, the meso/family and community level, and the macro/society, state and health system level. The result is a matrix of factors that explain the particular vulnerability of the study population during a pandemic. The authors add an important dimension by including ‘sources of resilience’ as factors that can influence the other elements within the framework. These sources of resilience can also be seen as potential policy entry points, as they highlight ways to mitigate not only the adverse effects of the pandemic itself but also the unintended consequences of pandemic policies. The sources of resilience are also categorised at the individual, community and societal levels and include examples such as psychological framing, recreational and spiritual activities, support from local communities, decent working conditions, access to health information, legal status and trust in authorities.

The literature review by Irizar and colleagues [36] uses an adapted version of the Diderichsen model – the Priority Public Health Conditions (PPHC) analytical framework – to examine the specific vulnerabilities of ethnic minority groups and Indigenous Peoples during the COVID-19 pandemic. Similar to the study by Hintermeier and colleagues, the authors present a more elaborated and detailed version of the framework, adapted to the context of an infectious viral disease. First, the authors apply the mechanisms of the PPHC model (socioeconomic context and position, differential exposure, differential vulnerability, differential health outcomes and differential social outcomes) to identify risk factors for two specific outcomes: infection and severe disease. Second, the authors adopt a similar structure to that in the previous review, categorising factors at the individual, community and societal levels. However, they also introduce a temporal dimension, distinguishing between three stages: before infection, during acute infection and after acute infection. A clear pattern emerges: prior to infection, exposure to the virus is the key factor, whereas vulnerability and social consequences become more important during and after illness. The authors emphasise, however, that an individual’s socioeconomic position plays a role across all three stages and at every level of analysis. The framework is therefore comparable to other theories of health inequality, such as fundamental cause theory, which argues that socioeconomic position has a flexible and multifaceted influence on both susceptibility to and resilience against illness and disease [8].

A third development of the Diderichsen model is presented by Katikireddi and colleagues [49], whose essay is aimed at outlining a framework for understanding the pathways that generate ethnic and racial inequalities in the context of COVID-19. Building on the Diderichsen model, the authors propose an expanded framework that, in addition to the familiar mechanisms of exposure, vulnerability and consequences, also incorporates two additional dimensions: differential effectiveness of control measures and differential adverse consequences of those measures. In this formulation, the pandemic is situated within a broader reality shaped not only by the virus itself and the environment in which it is spread, but also by political decision-making. The concept of differential effectiveness refers to how well control measures are tailored to protect different segments of the population, as well as how receptive various groups are to public health campaigns and vaccine offers. These variations are often linked to historical experiences with public health interventions and levels of trust in institutions. The dimension of differential adverse consequences captures the fact that some groups are better equipped than others to cope with the social and economic fallout of pandemic responses. In addition, the framework draws attention to the complexity and often double-edged nature of pandemic responses. While policy entry points aim to reduce exposure and vulnerability, they can also introduce measures that unintentionally disadvantage certain groups. Taken together, the mechanisms outlined in this framework provide insight into the disproportionate burden of the pandemic experienced by ethnic minority populations, which is a pattern that has been documented in numerous countries around the world [35].

## Discussion

This review examined how the Diderichsen model has been used to analyse health inequalities during the COVID-19 pandemic. While most studies cited the model to establish a theoretical foundation, a smaller number applied it more directly to interpret empirical findings or develop adapted frameworks. Among the studies that referred to the Diderichsen model as part of the article’s background or discussion, two main patterns emerged: first, the model was used as a way to position the study within the broader field of health inequality research; second, the model’s core mechanisms – differential exposure, vulnerability and consequences – served as a common analytical structure for explaining why socially disadvantaged groups were disproportionately affected by the pandemic.

Many studies emphasised how exposure was shaped by factors such as occupational roles and housing conditions, while vulnerability was linked to age, comorbidities and limited access to healthcare and vaccination. Several studies also explored the cascading social consequences of the pandemic, including job loss, reduced access to essential services and rising poverty. In addition, a few articles proposed expanded versions of the model, incorporating temporal dimensions, multilevel structures, and resilience factors, highlighting its adaptability for future research on pandemic-related health inequalities. While the studies reviewed here did not quantify these pathways in clinical terms, they illustrate how social, economic and political contexts determine the conditions under which risk accumulates and persists. This underlines the need to understand health inequalities as the result of interactions between social disadvantage and biological risk. Closer collaboration between social science and clinical research could help to address these intertwined pathways more effectively and ensure that different disciplinary perspectives inform both research and policy.

The articles that further developed the model did so with a focus on the particularly vulnerable situation of migrants and ethnic minorities during the pandemic. This focus is understandable, as this dimension of inequality has been especially significant in relation to the burden of COVID-19 in many countries. The studies developed the Diderichsen model by suggesting new mechanisms of inequality related to the pandemic measures [49], categorising the mechanisms into micro, meso and macro levels [35] and introducing a temporal dimension differentiating between inequality taking place before, during or after the pandemic [36]. However, these further-developed models did not explicitly engage with social justice as a distinct analytical or normative dimension. While they acknowledged that inequalities result from ‘modifiable social arrangements’, using the words by Diderichsen and colleagues, they did not fully explore how these inequalities relate to broader questions of fairness, inclusion, prioritisation and discrimination. In particular, the models did not address how assumptions about who is seen as deserving of protection may influence both the design and the impact of pandemic responses. Although the focus on mechanisms and pathways is highly valuable, there remains an opportunity to further develop the model by incorporating a more explicit consideration of social justice, including the normative assumptions that guide decisions about whose health should be protected, and on what grounds.

### Who is worthy of protection? Politics of recognition and redistribution in pandemic times

In New York, Rome, Madrid and Stockholm, people stood on their balconies in the spring evening light and applauded into the empty air. A scene that would have seemed inexplicable a year earlier quickly became a symbol of the gratitude many felt for healthcare workers who risked their lives to care for critically ill patients in the early stages of the pandemic. These repeated rounds of applause were clear acts of *recognition*, which have proven to be important in the context of several infectious diseases. Conversely, the absence of recognition has had serious consequences for the response to previous epidemics. A striking example is the fact that President Ronald Reagan did not publicly mention AIDS by name until four years into an epidemic that had already claimed thousands of lives in the United States alone [50]. This silence was in all likelihood linked to the Reagan administration’s reluctance to associate itself with gay men and other stigmatised groups affected by HIV/AIDS, and had real consequences for work against the disease [51]. Would research have been more advanced, would the development of antiretroviral drugs have been faster, would we even have a cure today, if HIV had affected communities closer to the centre of power? The WHO asks similar questions through addressing the insufficient recognition of health issues classified as ‘neglected tropical diseases’ [52]. These include infectious diseases that primarily affect impoverished communities in tropical regions, such as dengue, schistosomiasis, leishmaniasis and yaws. Although they affect more than a billion people worldwide, such diseases are frequently absent from the global health agenda and often overlooked by major funding bodies.

This last point also highlights the close relationship between recognition and economic redistribution. Issues that are placed high on the political agenda and receive significant public attention tend to be prioritised in terms of financial support. The extraordinary attention given to COVID-19 undoubtedly contributed to the decisive response to the pandemic and the rapid development of effective vaccines. However, not everyone benefitted from this attention. Indeed, a central point of Nancy Fraser’s article is that cultural recognition has replaced economic redistribution as the main mode of social justice. Applause from the balcony costs nothing; while many healthcare workers expressed appreciation for the public sympathy, they also emphasised that this intangible recognition must be translated into concrete support – such as access to protective equipment, improved working conditions and fair economic compensation [53]. This raises a critical question: for which groups within a given country does recognition actually translate into prioritisation in the distribution of health-promoting interventions? Fraser’s theory of social inequality, which points to the interplay of economic and cultural injustice, provides a useful basis for extending the Diderichsen model. Particularly when a public health issue is shaped not only by ‘exposure’ and ‘vulnerability’ but also by political decision-making, the question of health inequality becomes one of both material redistribution and symbolic recognition. In this way, the pandemic did not merely serve as a test case for the model but also revealed certain conceptual gaps, most notably the lack of an explicit dimension addressing social recognition and perceived worthiness. In such contexts, the question ‘who is worthy of protection?’ gains urgent and tangible significance, especially during a deadly pandemic, as it reflects a form of formalised social recognition, encompassing both material and immaterial privileges. These might include the right to work from home, eligibility for economic compensation schemes, and prioritisation in the distribution of essential goods and services such as intensive care and vaccines. As such, the political and social perception of ‘worthiness’ is not merely a rhetorical question but can be understood as a conceptual addition that complements Diderichsen’s categories of ‘exposure’ and ‘vulnerability’ in a meaningful way.

The vaccine prioritisation strategies adopted by various countries were generally shaped by the same interplay between exposure and vulnerability that the Diderichsen model outlines. As a result, highly exposed groups, such as healthcare workers, were prioritised alongside vulnerable populations, including the elderly and individuals with underlying health conditions [54]. Another group frequently highlighted in national vaccination programmes was so-called ‘essential workers’. This category includes not only healthcare personnel, but also police officers, firefighters, utility workers, senior government officials, border guards, customs officers and members of the armed forces [55]. Several interrelated factors likely contribute to the prioritisation of these groups. In some cases, their inclusion may be justified by a heightened risk of exposure to the virus. In others, the emphasis is placed on the critical societal functions they perform. It is likely that a dimension of social status or cultural recognition also plays a role, helping to elevate these groups on the prioritisation lists.

Studies on public attitudes towards prioritisation strategies also reflect broader views on who is considered worthy of protection. A public survey conducted in the United States showed widespread support for the official prioritisation framework, particularly highlighting the importance of vaccinating healthcare workers, but also recognising the need to prioritise racial and ethnic minorities disproportionately affected by COVID-19 [56]. In some cases, however, prioritisation programmes overlooked factors related to particular exposure or vulnerability. One study, for instance, found that only a minority of US states prioritised the vaccination of incarcerated individuals, despite their classification as a highly exposed group, whereas a significantly larger number prioritised correctional staff [57]. In Sweden, the Public Health Agency removed undocumented migrants from the list of groups to be prioritised on the basis of social vulnerability. This decision had been preceded by public criticism, including from Sweden’s current foreign minister, who described the agency’s initial prioritisation as ‘unreasonable’ and argued that ‘non-compliance [with deportation orders] should not be rewarded’ [58].

## Conclusion

This review shows that the Diderichsen model continues to serve as a key framework in the study of health inequalities, including during the COVID-19 pandemic. While its core mechanisms – exposure, vulnerability and social consequences – remain widely relevant, the pandemic context has prompted new developments. Several studies applied the model to analyse how social disadvantage shaped pandemic outcomes, while others expanded the framework to include new dimensions such as the effectiveness and adverse effects of control measures, multilevel structures and temporal phases of inequality. Across the literature, there is a clear recognition of how structural factors, such as class, ethnicity and legal status, influenced both direct health risks and broader social consequences. Drawing on Fraser’s theory of justice, the review argues that the pandemic makes visible the role of both material distribution and symbolic recognition in shaping health outcomes. The extent to which groups are recognised as deserving of protection is a matter not only of risk but of political judgement.

## References

[1] Smith Jervelund S De Montgomery CJ . Nordic registry data: Value, validity and future. Scand J Public Health 2020;48:1-4. 10.1177/1403494819898573 31985364

[2] Mackenbach JP. Nordic paradox, Southern miracle, Eastern disaster: Persistence of inequalities in mortality in Europe. Eur J Public Health 2017;27:14-7. 10.1093/eurpub/ckx160 29028239

[3] Diderichsen F Evans T Whitehead M . The social basis of disparities in health. In: Evans T (ed.) Challenging inequalities in health: From ethics to action. New York, NY: Oxford University Press, 2001, pp.13-23.

[4] Dyar OJ Haglund BJA Melder C , et al. Rainbows over the world’s public health: Determinants of health models in the past, present, and future. Scand J Public Health 2022;50:1047-58. 10.1177/14034948221113147 PMC9578089 36076363

[5] Diderichsen F Hallqvist J Whitehead M. Differential vulnerability and susceptibility: How to make use of recent development in our understanding of mediation and interaction to tackle health inequalities. Int J Epidemiol 2018;48:268-74. 10.1093/ije/dyy167 30085114

[6] Solar O Irwin A . A conceptual framework for action on the social determinants of health. Geneva: WHO Document Production Services, 2010.

[7] Krieger N. Theories for social epidemiology in the 21st century: An ecosocial perspective. Int J Epidemiol 2001;30:668-77. 10.1093/Ije/30.4.668 11511581

[8] Link BG Phelan J . Social conditions as fundamental causes of disease. J Health Soc Behav 1995:80-94. 7560851

[9] Scholthof K-BG . The disease triangle: Pathogens, the environment and society. Nat Rev Microbiol 2007;5:152-6. 10.1038/nrmicro1596 17191075

[10] Fraser N . From redistribution to recognition?: Dilemmas of justice in a ‘postsocialist’ age. In: Seidman S and Alexander JC (ed.) The new social theory reader. London: Routledge, 2020, 2nd edition pp.188-96.

[11] Mein SA. COVID-19 and health disparities: The reality of “the great equalizer”. J Gen Intern Med 2020;35:2439-40. 10.1007/s11606-020-05880-5 PMC7224347 32410124

[12] Clouston SA Natale G Link BG. Socioeconomic inequalities in the spread of coronavirus-19 in the United States: A examination of the emergence of social inequalities. Soc Sci Med 2021;268:113554. 10.1016/j.socscimed.2020.113554 PMC7703549 33308911

[13] Beese F Waldhauer J Wollgast L , et al. Temporal dynamics of socioeconomic inequalities in COVID-19 outcomes over the course of the pandemic—a scoping review. Int J Public Health 2022;67:1605128. 10.3389/ijph.2022.1605128 PMC9464808 36105178

[14] Eriksson S. Så spred “Alpernas Ibiza” viruset i Europa. Svenska Dagbladet 19 March 2020.

[15] Gauffin K Östergren O Cederström A. Waves of inequality: Income differences in intensive care due to Covid-19 in Sweden. Eur J Public Health 2023;33:574-9. 2023/06/16. 10.1093/eurpub/ckad094 PMC10393505 37322545

[16] Mutambudzi M Niedzwiedz C Macdonald EB , et al. Occupation and risk of severe COVID-19: Prospective cohort study of 120 075 UK Biobank participants. Occup Environ Med 2021;78:307. 10.1136/oemed-2020-106731 PMC7611715 33298533

[17] McNamara CL McKee M Stuckler D. Precarious employment and health in the context of COVID-19: A rapid scoping umbrella review. Eur J Public Health 2021;31:iv40-9. 10.1093/eurpub/ckab159 PMC8576296 34751369

[18] Hayward SE Deal A Cheng C , et al. Clinical outcomes and risk factors for COVID-19 among migrant populations in high-income countries: A systematic review. J Migr Health 2021;3:100041. 10.1016/j.jmh.2021.100041 PMC8061095 33903857

[19] Mackey K Ayers CK Kondo KK , et al. Racial and ethnic disparities in COVID-19–related infections, hospitalizations, and deaths. Ann Intern Med 2020;174:362-73. 10.7326/M20-6306 PMC7772883 33253040

[20] Williams DB Spinks B Williams D , et al. Effects of the COVID-19 pandemic on people experiencing incarceration: A systematic review. BMJ Open 2024;14:e076451. 10.1136/bmjopen-2023-076451 PMC11002388 38582532

[21] Mohsenpour A Bozorgmehr K Rohleder S , et al. SARS-Cov-2 prevalence, transmission, health-related outcomes and control strategies in homeless shelters: Systematic review and meta-analysis. EClinicalMedicine 2021;38:101032. 10.1016/j.eclinm.2021.101032 PMC8298932 34316550

[22] Whitehead M Barr B Taylor-Robinson D. Covid-19: We are not “all in it together”—less privileged in society are suffering the brunt of the damage. thebmjopinion 2020. https://blogs.bmj.com/bmj/2020/05/22/covid-19-we-are-not-all-in-it-together-less-privileged-in-society-are-suffering-the-brunt-of-the-damage/

[23] Sukhera J. Narrative reviews: Flexible, rigorous, and practical. J Grad Med Educ 2022;14:414-7. 10.4300/JGME-D-22-00480.1 PMC9380636 35991099

[24] Ferrari R. Writing narrative style literature reviews. Med Writing 2015;24:230-5.

[25] Diderichsen F Andersen I Manuel C , et al. Health inequality – determinants and policies. Scand J Public Health 2012;40:12-105. 10.1177/1403494812457734 23147863

[26] Diderichsen F Hallqvist J . Social inequalities in health: Some methodological considerations for the study of social position and social context.In: Arve-Parès B (ed.) Inequality in health: A Swedish perspective. Stockholm: Swedish Council for Social Research, 1998, pp.25-39.

[27] Antequera A Lawson DO Noorduyn SG , et al. Improving social justice in COVID-19 health research: Interim guidelines for reporting health equity in observational studies. Int J Environ Res Public Health 2021;18:9357. 34501949 10.3390/ijerph18179357 PMC8431098

[28] Den Broeder L South J Rothoff A , et al. Community engagement in deprived neighbourhoods during the COVID-19 crisis: Perspectives for more resilient and healthier communities. Health Promot Int 2022;37:daab098. 10.1093/heapro/daab098 PMC8414056 34297841

[29] Demenech LM Dumith SdC Vieira MECD , et al. Income inequality and risk of infection and death by COVID-19 in Brazil. Rev Bras Epidemiol 2020;23:e200095. 10.1590/1980-549720200095 33027434

[30] Diderichsen F Andersen I Mathisen J. Depression and diabetes: The role of syndemics in the social inequality of disability. J Affect Disord Rep 2021;6:100211.

[31] Diderichsen F. How did Sweden fail the pandemic? Int J Health Serv 2021;51:417-22. 10.1177/0020731421994848 PMC8436291 33635123

[32] Foster HM Ho FK Mair FS , et al. The association between a lifestyle score, socioeconomic status, and COVID-19 outcomes within the UK Biobank cohort. BMC Infect Dis 2022;22:273. 35351028 10.1186/s12879-022-07132-9 PMC8964028

[33] Green MJ Craig P Demou E , et al. Understanding inequalities in mental health by family structure during COVID-19 lockdowns: Evidence from the UK household longitudinal study. Ann Gen Psychiatry 2023;22:24. 37280641 10.1186/s12991-023-00454-1 PMC10242239

[34] Hanson S Belderson P Ward E , et al. Lest we forget. Illuminating lived experience of the Covid-19 pandemic and lockdown. Soc Sci Med 2023;332:116080. 37451941 10.1016/j.socscimed.2023.116080

[35] Hintermeier M Gottlieb N Rohleder S , et al. COVID-19 among migrants, refugees, and internally displaced persons: systematic review, meta-analysis and qualitative synthesis of the global empirical literature. EClinicalMedicine 2024;74:102698. 10.1016/j.eclinm.2024.102698 PMC11701484 39764176

[36] Irizar P Pan D Taylor H , et al. Disproportionate infection, hospitalisation and death from COVID-19 in ethnic minority groups and Indigenous Peoples: An application of the Priority Public Health Conditions analytical framework. EClinicalMedicine 2024;68:102360. 10.1016/j.eclinm.2023.102360 PMC10965404 38545088

[37] Lingam L Suresh Sapkal R. COVID-19, physical distancing and social inequalities: Are we all really in this together? Int J Community Soc Dev 2020;2:173-90.

[38] Marra M Strippoli E Zengarini N , et al. Inequalities in the health impact of the first wave of the COVID-19 pandemic in Piedmont Region, Italy. Int J Environ Res Public Health 2022;19:14791. 36429508 10.3390/ijerph192214791 PMC9690941

[39] McGowan VJ Bambra C. COVID-19 mortality and deprivation: Pandemic, syndemic, and endemic health inequalities. Lancet Public Health 2022;7:e966-75. 10.1016/S2468-2667(22)00223-7 PMC9629845 36334610

[40] Netrdová P Tesárková KH Dzúrová D. Exploring vulnerability amplification in regional health inequality: COVID-19 case study in Czechia. Appl Geogr 2025;177:103565.

[41] Pattanshetty S Pardesi M Gudi N. A comparative analysis on the social determinants of COVID-19 vaccination coverage in fragile and conflict affected settings and non-fragile and conflict affected settings. Int J Health Policy Manage 2022;12:6830. 10.34172/ijhpm.2022.6830 PMC10125044 36300252

[42] Paykani T Oana I-E. Sociopolitical context and COVID-19 fatality rates in OECD countries: A configurational approach. BMC Public Health 2024;24:2400. 39232770 10.1186/s12889-024-19594-4 PMC11373141

[43] Rigó M Weyers S. Child motor development before and after the COVID-19 pandemic: Are there social inequalities? Children 2024;11:936. 39201871 10.3390/children11080936 PMC11353027

[44] Sandholdt CT Srivarathan A Kristiansen M , et al. Undertaking graphic facilitation to enable participation in health promotion interventions in disadvantaged neighbourhoods in Denmark. Health Promot Int 2022;37:ii48-59. 10.1093/heapro/daac034 PMC9384324 35748284

[45] Larsen TS Halberg N Jensen PS , et al. Emotional risk work during the pandemic: Healthcare professionals’ perceptions from a COVID-19 ward. Health, Risk Soc 2023;25:110-28.

[46] Schrecker T. The COVID-19 Pandemic as a tipping point: What future for the right to health? Health Hum Rights 2023;25:111. 38145142 PMC10733769

[47] Vignola EF Andrea SB Hajat A , et al. What extraordinary times tell us about ordinary ones: A multiple case study of precariously employed food retail and service workers in two US state contexts during the COVID-19 pandemic. J Crit Public Health 2024;1:45. 39239638 10.55016/ojs/jcph.v1i1.78291 PMC11376689

[48] Ying Y-H Lee W-L Chi Y-C , et al. Demographics, socioeconomic context, and the spread of infectious disease: The Case of COVID-19. Int J Environ Res Public Health 2022;19:2206. 35206390 10.3390/ijerph19042206 PMC8872250

[49] Katikireddi SV Lal S Carrol ED , et al. Unequal impact of the COVID-19 crisis on minority ethnic groups: A framework for understanding and addressing inequalities. J Epidemiol Community Health 2021;75:970-4. 10.1136/jech-2020-216061 PMC8458062 33883198

[50] Curran JW Jaffe HW Hardy AM , et al. Epidemiology of HIV infection and AIDS in the United States. Sci 1998;239(4840):610-616. 10.1126/science.3340847 3340847

[51] Padamsee TJ . Fighting an epidemic in political context: thirty-five years of HIV/AIDS policy making in the United States. Soc Hist Med 2020;33(3):1001-1028. 33424441 10.1093/shm/hky108 PMC7784248

[52] Hotez PJ Aksoy S Brindley PJ , et al. What constitutes a neglected tropical disease?: San Francisco: Public Library of Science, 2020, p.e0008001. 10.1371/journal.pntd.0008001 PMC6991948 31999732

[53] Djupenström H. Vårdpersonal: Sluta applådera – och hjälp till. Svenska Dagbladet, 24 March 2020.

[54] Cylus J Panteli D Van Ginneken E. Who should be vaccinated first? Comparing vaccine prioritization strategies in Israel and European countries using the Covid-19 Health System Response Monitor. Isr J Health Policy Res 2021;10:1-3. 33608023 10.1186/s13584-021-00453-1 PMC7893378

[55] Straetemans M Buchholz U Reiter S , et al. Prioritization strategies for pandemic influenza vaccine in 27 countries of the European Union and the Global Health Security Action Group: A review. BMC Public Health 2007;7:1-12. 17825095 10.1186/1471-2458-7-236 PMC2048949

[56] Persad G Emanuel EJ Sangenito S , et al. Public perspectives on COVID-19 vaccine prioritization. JAMA Netw Open 2021;4:e217943. 10.1001/jamanetworkopen.2021.7943 PMC8035644 33835172

[57] Strodel R Dayton L Garrison-Desany HM , et al. COVID-19 vaccine prioritization of incarcerated people relative to other vulnerable groups: An analysis of state plans. PLoS One 2021;16:e0253208. 10.1371/journal.pone.0253208 PMC8205184 34129620

[58] Andersson L. FHM ändrar vaccinprioritet – tar bort ”papperslösa”. Omni, 5 February 2021.

[59] Coleman P Barber TM Van Rens T , et al. COVID-19 outcomes in minority ethnic groups: do obesity and metabolic risk play a role? Curr Obes Rep 2022; 11: 107-115. 34655051 10.1007/s13679-021-00459-5 PMC8518892

